# Oral maxillary exostosis

**DOI:** 10.1002/ccr3.1918

**Published:** 2018-11-11

**Authors:** Luisa Limongelli, Angela Tempesta, Saverio Capodiferro, Eugenio Maiorano, Gianfranco Favia

**Affiliations:** ^1^ Department of Interdisciplinary Medicine Aldo Moro University Bari Italy; ^2^ Department of Emergency and Organ Transplantation Aldo Moro University Bari Italy

**Keywords:** benign bone lesions, maxillary exostosis

## Abstract

Oral maxillary exostoses are proliferating bone lesions with an unknown etiology occurring on the cortical plates both in the maxilla and in the mandible of young individuals, showing a typical slow but continuous enlargement. No treatment is usually required unless they create esthetic or functional limitations during follow‐up; the biopsy is needed only for doubtful lesions. Furthermore, it is mandatory to collect an accurate familiar history of patients affected by exostosis, especially when occurring with atypical clinical presentation, in order to exclude or prevent potentially associated systemic diseases.

A 50‐year‐old man was referred at the outpatient clinic for multiple hard masses of the maxilla. The intraoral examination revealed multiple proliferating lesions of hard consistency, painless, and covered by normally colored mucosa, in the buccal aspect of the maxilla above the teeth (Figure 1). A slow but steady dimensional increasing was referred by the patient over the last year. The familial medical history was negative for bowel disease and for similar jaw lesions. On the panoramic radiograph (Figure 2), multiple well‐defined radiopacities with a round/ovoid appearance were detectable over the upper jaw. The cone beam computed tomography (Figure 3A‐E) revealed multiple proliferating osseous lesions with irregular appearance emerging from the buccal cortical plate of the maxilla without signs of teeth involvement. The diagnosis was of typical multiple buccal exostosis of the maxilla; a periodical follow‐up was suggested.

**Figure 1:**
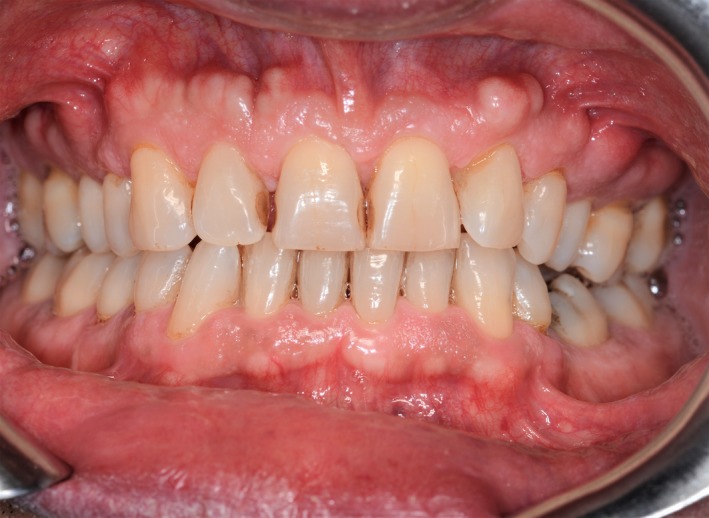
Multiple proliferating lesions of hard consistency in the buccal aspect of the maxilla above the teeth covered by normally colored mucosa

**Figure 2:**
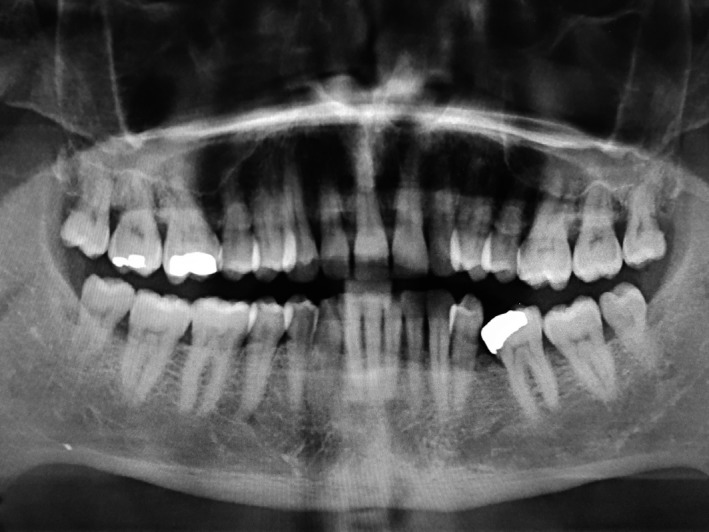
Panoramic radiograph showing multiple well‐defined radiopacities with a round/ovoid appearance all over the upper jaw

**Figure 3:**
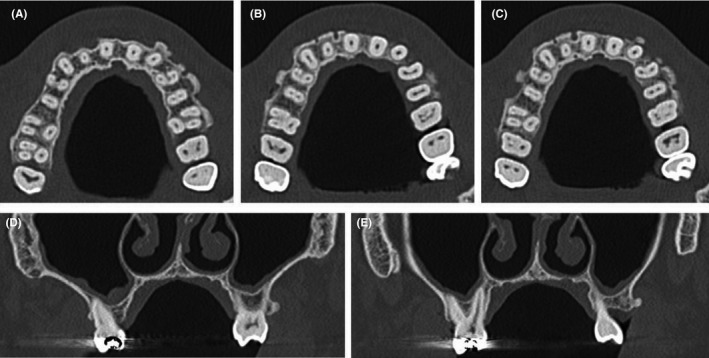
(A‐E) The cone beam computed tomography showed multiple proliferating osseous lesions with irregular appearance and dimension emerging from the buccal cortical plate of the maxilla without signs of teeth involvement

Oral exostoses (OEs) are bony protuberances arising from the buccal or lingual cortical plates of the maxilla and/or the mandible, with a variable prevalence ranging from 8% to 51% in the maxilla and 6%‐32% in the mandible.^1^ Although theories regarding a possible role of a chronic periosteal inflammation have been suggested, the true etiology of OE remains still unclear.^1^, ^2^ Though the appearance reported here is typical of oral exostosis, benign and malignant bone tumors should be considered in the differential diagnosis when the lesion presents as solitary localized enlargement. In addition, such patients should be investigated for Garden's syndrome by an accurate familial anamnesis and subsequently by colonoscopy in doubtful cases, considering the high rate of malignant transformation of intestinal polyps.

## CONFLICT OF INTEREST

Authors declare no conflict of interest.

## AUTHOR CONTRIBUTION

LL: performed clinical diagnosis and prepared the manuscript. AT: reviewed the literature. SC: performed clinical diagnosis and prepared the manuscript. EM: contributed to the final revision of the manuscript. GF: performed clinical diagnosis and prepared the manuscript.
